# Annotations

**Published:** 1899-05-27

**Authors:** 


### ANNOTATIONS.

### Scarlet Fever Spread by Milk.

The city of Buffalo in the United States has recently suffered from a small outbreak of scarlet fever which serves to illustrate again the ease with which the infection of that disease may be distributed by means of milk. We learn from the New York *Medical News* that, within three days in February, 21 cases of scarlet fever were reported from a certain section of Buffalo. They all occurred along a single milkman's route. Investigation showed that everything was in satisfactory condition as far as the milkman himself and his family were concerned, and also that the city milk dealer's premises and help were above suspicion. One of the farmers, however, who supplied the dealer with milk was found to be convalescent from some disease, and although the exact nature of this disease had not been determined, it was found that four other members of his family were suffering from undoubted scarlet fever. On this being discovered no more of this farmer's milk was allowed to be sent to the city, and the spread of the epidemic immediately stopped, the only fresh cases that occurred after this date being reported from families already infected with the disease. This little outbreak, apparently so definitely connected with the milk supply, is of interest in connection with the report by Dr. Klein on the microbes associated with scarlet fever, which appeared in the last report of the medical officer to the Local Government Board,* in which it was shown that the bacillus which has recently been shown to occur in connection with scarlet fever is the same as that which Dr. Klein some years ago demonstrated on the teats and udders of certain cows which had been convicted of disseminating that disease.

* See The Hospital, April 1st, 1899, page 3.

### Famine and Infant Mortality.

It is generally supposed that when there is a dearth of food the children die first. They are weaker, can less well stand privation or the substitution of food hard of digestion for that to which they are accustomed. If statistics do not bear out this view, the fact is usually attributed to the devotion of parents, who stint themselves rather than let their children languish. There is no doubt that this is true, and that a parent may practically starve to death by the side of a comparatively well-fed child. But it is curious that in times of famine infants, the most delicate portion of the population, that portion whose rate of mortality is always highest, seem to survive, nay, even to thrive. If statistics be necessary to support this astonishing fact, they may easily be found. During the cotton famine in Lancashire the general mortality was nearly double that of average times, but the death-rate of infants, that is, of children under one year, was reduced by 46 per cent. During the siege of Paris the same thing was observed; the death-rate among infants was only half as great as usual. What reason can be assigned for such facts as these? Dr. Templeman, the medical officer of health for Dundee, traces it to the fact that during such times infants are more likely to get the food nature provides for them. In Lancashire a great number of women are engaged in the factories, and their children are brought up, not very carefully in many cases, by hand. In France the suckling of children is very unusual, except amongst those persons who can afford a wet nurse. But, in famine time, mother's milk is one of the few commodities that can be got for nothing; therefore, in the dire stress of the situation, mothers who, either for work or pleasure, would have been absent from their children, leaving them to the care of hirelings, were compelled, from sheer poverty, to nurse them themselves. And so the babies throve. Such facts as these bring forward very impressively the fact that hand-feeding is a dangerous thing, especially among the poor. Those who can afford experienced nurses, and whose homes make it possible that feeding-bottles wil be kept scrupulously clean, that food will be given at the right temperature, that milk will be sweet and pure, may attempt hand-feeding without evil results. But among the poor these advantages must be the exception rather than the rule. Therefore, it is of the first importance to impress upon poor mothers—who are getting lazy and selfish like their betters—the importance of suckling their children. Perhaps, however, there is another reason why infants do well in famine time. The poor are notoriously fond of giving their children "a bit of what's going," and what is going in their homes is often rather tasty than digestible. There is no doubt that children do better when mother's milk is their only fare, without the addition of appetising morsels of red herring, sausages, and the like.

### A Vestry's Perversity.

Of all the mean and futile methods of inflicting annoyance on a hard-working public official commend us to that adopted by the Vestry of St. George's, Southwark, in dealing with the report of Dr. Waldo, their medical officer of health. This report is drawn up, is printed, and is presented to the Sanitary Committee, and in the ordinary course it should now be accessible to the public, and, so far as it is open to an enthusiastic medical officer to gain credit among his fellows by showing a good record of work well done, Dr. Waldo ought now to be enjoying the gratification of displaying to the medical officers of health of other districts what he has been doing in the somewhat dreary area which he has under his care. But no! It seems to have occurred to this sapient vestry that by refusing to publish the report, by having only a limited number printed, and by not issuing even those until some distant date, when they can be bound up with the annual report of the vestry, they could at the same time annoy their officer and perchance hide from the public some of the sanitary deficiencies of their delightful district—its high death-rate, its high infantile mortality, its excessive prevalence of zymotic disease, its insanitation, its overcrowding, and, to crown all, the obstinate refusal of its vestry to obey the orders of the Home Office to clear the crypt of its parish church of the thousand coffins and dead bodies which it contains. Vain fancy! Dr. Waldo still goes about his district undisturbed, while as for the attempt to suppress the publication of the truth about their district, the local papers publish full extracts from the report just as if the vestry had never laid any embargo at all upon its issue! Muzzling the press is not so easy a performance as these good men of Southwark seem to imagine.

